# Manganese-Enriched Biochar Reduces Cd Uptake and Accumulation in Rice by Altering Soil Cd Speciation and Enhancing Mn–Cd Antagonism

**DOI:** 10.3390/toxics14040346

**Published:** 2026-04-20

**Authors:** Qian Wang, Xu Yan, Kexin Shao, Lingfei Zuo, Haoran Jiao, Wenjuan Fan, Juan Lin, Jinbiao Li, Min Lv, Anyong Hu, Yujie Han

**Affiliations:** School of Geographical Science, Nantong University, Nantong 226019, China; 15052135695@163.com (Q.W.); yanxu20050129@sina.com (X.Y.); skx20040414@sina.com (K.S.); 13306186150m@sina.cn (L.Z.); 19732370972@sina.cn (H.J.); 2686fan@sina.com (W.F.); linjuan@ntu.edu.cn (J.L.); jbli@ntu.edu.cn (J.L.); lm2018@ntu.edu.cn (M.L.)

**Keywords:** paddy soil, Cd contamination, Mn-enriched biochar, Cd bioavailability, Cd accumulation

## Abstract

Cadmium (Cd) contamination in paddy soils threatens rice production and food safety. This study investigated the effects of manganese (Mn)-enriched biochar on soil Cd immobilization and Cd accumulation in rice using a pot experiment with Cd-contaminated soil. Unenriched biochar and Mn-enriched biochar prepared from rice straw were applied at two rates (0.5% and 1.0%). Both biochar types significantly increased soil pH and organic matter and promoted the transformation of Cd from labile fractions to more stable residual forms, thereby reducing Cd bioavailability. As a result, Cd accumulation in rice tissues, including straw and brown rice, was significantly reduced. Correlation analysis further indicated that increased soil pH was associated with reduced Cd mobility and plant uptake. Mn-enriched biochar markedly increased Mn accumulation and uptake efficiency in rice while decreasing Cd uptake efficiency, indicating a strong antagonistic interaction between Mn and Cd in the soil–plant system. Notably, a low application rate of Mn-enriched biochar (0.5%) achieved Cd reduction effects comparable to those of a higher dose of unenriched biochar (1.0%). These results suggest that Mn-enriched biochar is an effective and potentially cost-efficient strategy for reducing Cd bioavailability in paddy soils and mitigating Cd accumulation in rice.

## 1. Introduction

With the rapid advancement of industrialization in China, land pollution issues caused by industrial production and mineral resource exploitation have also been increasingly exacerbated. According to the 2014 “National Soil Pollution Status Survey Report of China,” the rate of soil points exceeding standard levels nationwide has reached 16.1%, with cadmium (Cd) contamination being the most prevalent among inorganic pollutants, with a point exceedance rate of 7.0% [1]. The pollution level in southern China is significantly higher than in the north. Furthermore, the contamination of arable land is particularly severe, with an exceedance rate of 19.4% [2]. As a major producer and consumer of rice, China’s rice yield and quality are directly linked to national food security and the health of its people. Cadmium (Cd) is a highly toxic heavy metal and a non-essential element for plant growth and development. When exposed to Cd-contaminated soil, plants absorb Cd through their roots. As it accumulates within the plant and reaches a certain threshold, it causes various toxic symptoms, such as leaf chlorosis, shortened stems, reduced lateral roots, inhibited chlorophyll synthesis, decreased antioxidant enzyme activity, and increased cell membrane permeability, leading to cell damage and even death. This, in turn, suppresses the normal growth and development of plants [3,4]. In agricultural soils, heavy metal Cd can accumulate through the food chain [5], causing damage to major human organs such as the bones, liver, and kidneys. It can further impair the immune system and, in severe cases, lead to cancer, birth defects, and other health risks [6].

A wide range of remediation technologies, including in situ and ex situ approaches, have been employed to address soil contamination. Among them, in situ methods are generally preferred due to their reduced risk of human exposure, lower environmental disturbance, and improved cost efficiency [7]. Although physicochemical techniques have demonstrated high remediation effectiveness, their practical application is often constrained by high operational costs, the risk of secondary pollution, and potential degradation of soil quality and microbial communities [8]. As a result, biological approaches, particularly phytoremediation, have emerged as more sustainable and economically viable alternatives for restoring contaminated soils. However, phytoremediation is often limited by the long time required for effective heavy metal removal and the challenge of safely disposing of contaminated biomass generated during phytoextraction, which may pose risks of secondary environmental pollution [9,10]. Biochar is a carbon-rich solid product obtained by pyrolyzing wood, crop straw, and other biomass under high-temperature, oxygen-limited conditions. The primary elemental composition of biochar includes C, H, and O, and depending on the raw materials used, its ash content may also contain nutrients such as N, P, K, Ca, Na, and Mg [11,12]. In recent years, numerous studies have shown that biochar has significant potential for the remediation of contaminated agricultural soils. Its main mechanisms of soil remediation include: increasing soil pH, enhancing organic matter content, boosting cation exchange capacity, improving soil physical properties (such as aeration and water retention), and enhancing soil aggregate structure. These effects collectively improve the soil’s physicochemical properties and overall nutrient status [13,14,15]. However, most studies have focused primarily on general physicochemical effects (e.g., pH increase and adsorption), and the remediation efficiency often depends on relatively high application rates. Manganese (Mn) is one of the essential trace elements for plant growth and development. It is primarily absorbed by plants in the form of Mn^2+^ and participates in various physiological processes within the plant, such as photosynthesis, antioxidant defense, and metal ion metabolism [16]. Recent studies have shown that Mn and Cd exhibit a significant antagonistic interaction in the soil–plant system. Specifically, Mn can compete with Cd for uptake pathways (e.g., via transporters such as OsNRAMP5), thereby reducing Cd absorption and translocation in plants [17,18]. Proper application of manganese fertilizers can increase the content of available Mn in the soil, which helps reduce the absorption and transport of Cd by plants, indicating that Mn has a clear inhibitory effect on Cd absorption [19,20,21]. Although Mn–Cd competition at the plant uptake level has been widely reported, recent studies highlight that Mn also plays an important role in controlling Cd mobility in soils, particularly through the formation of Mn oxides. On the one hand, Mn oxides can act as dominant carriers of Cd in soils, effectively immobilizing Cd through stable inner-sphere complexation (e.g., Cd–O and Cd–OH bonds), thereby reducing its mobility and bioavailability [22]. On the other hand, Mn application (e.g., MnSO_4_) significantly increases soil available Mn and promotes the formation of free and amorphous Mn oxides, thereby enhancing Cd binding to Mn oxides, reducing Cd bioavailability, and inducing shifts in the chemical speciation of both Cd and Mn [23]. These findings highlight the dual role of Mn in Cd immobilization in soil and inhibition of Cd accumulation in plants, providing a mechanistic basis for Mn-based remediation strategies. Although some studies have reported the effectiveness of Mn-modified biochar in immobilizing heavy metals [24,25,26], these studies are relatively limited and mainly emphasize soil adsorption and chemical transformations, with less attention given to plant uptake processes and overall remediation efficiency in soil–plant systems. Despite these advances, several critical knowledge gaps remain that need to be addressed. First, most studies emphasize soil Cd fraction transformation or adsorption mechanisms, while fewer studies systematically link soil chemical changes, Cd speciation, and plant uptake processes in an integrated soil–plant system. Second, existing studies rarely compare different Mn sources (e.g., MnCl_2_ vs. KMnO_4_) or evaluate whether Mn-enriched biochar can achieve comparable remediation efficiency at lower application rates, which is critical for practical and economic feasibility. Therefore, this study aimed to evaluate the effects of Mn-enriched biochar on soil Cd immobilization and Cd accumulation in rice, and to elucidate the underlying processes by linking biochar-induced changes in soil chemical properties and Cd fraction transformation with Cd immobilization and Mn–Cd antagonistic interactions in the soil–plant system, while further assessing whether Mn-enriched biochar can achieve comparable remediation efficiency at reduced application rates.

## 2. Materials and Methods

### 2.1. Plant Materials and Biochar Preparation

The soil used in the experiment was collected from the cadmium-contaminated plow layer (0–20 cm) of a paddy field in Guixi, Jiangxi Province, China. The soil is classified as a paddy soil developed from red soil (Ultisol). The rice variety used for the experiment was “Nipponbare.” The pot experiment was conducted under natural greenhouse conditions to simulate a typical paddy field environment. The preparation process for manganese-enriched biochar is as follows: rice straw was selected as the carbonization material, crushed, and placed into a tube furnace. It was pyrolyzed at 550 °C under nitrogen gas for 2 h to obtain the raw biochar. The rice straw-derived biochar had a pH of 9.85, with total carbon (TC) and total nitrogen (TN) contents of 45.6% and 0.62%, respectively. After grinding and sieving through a 100-mesh sieve, 100 g of uniform-sized biochar particles were soaked in 0.05 M and 0.10 M MnCl_2_ or KMnO_4_ solutions. MnCl_2_ and KMnO_4_ were selected to represent Mn sources with different oxidation states and reactivities, allowing the evaluation of how Mn speciation influences Cd immobilization and plant uptake. The mixture was stirred for 2 h using a magnetic stirrer and treated with 2 h of ultrasonic treatment to promote the uniform loading of Mn. After the treatment, the biochar was dried at 105 °C until constant weight was achieved. It was then ground in a mortar to obtain manganese-rich biochar, with Mn contents of approximately 27.5 mg Mn/g and 55 mg Mn/g of biochar, respectively.

### 2.2. Soil Pot Experiment Design

Soil samples passed through a 10-mesh sieve were weighed and mixed with different proportions and types of biochar according to the treatment groups. Specifically, 12.5 g (0.5%) and 25 g (1%) of biochar, both unenriched and manganese-enriched, were mixed uniformly with 2.5 kg of soil in large plastic bags. Subsequently, nitrogen, phosphorus, and potassium fertilizers (urea for N, sodium dihydrogen phosphate for P, and potassium chloride for K) were added to each mixture, ensuring that the applied amounts of N, P, and K were 100 mg/kg of soil. After thorough mixing, the samples were transferred to plastic pots, and water was added to moisten the soil. The pots were allowed to sit for 10 days to equilibrate. Rice seedlings that had been grown hydroponically for 20 days were then transplanted into the pots, with 4 plants per pot. A total of 11 treatment groups were set up: a control without biochar (CK); 0.5% and 1.0% unenriched biochar (BC1, BC2); and 0.5% and 1.0% of two Mn levels (27.5 mg Mn/g and 55 mg Mn/g) of MnCl_2_-enriched biochar (M1BC1, M2BC1, M1BC2, M2BC2) and KMnO_4_-enriched biochar (KM1BC1, KM2BC1, KM1BC2, KM2BC2). Each treatment had 3 replicates, with a total of 33 pots (Table 1).

### 2.3. Sample Collection and Preprocessing

After 105 days of growth following transplantation into the soil, at rice maturity, root, straw, rice grain, and soil samples were collected from each treatment group. The straw and rice grains were placed in a well-ventilated sunroom to air-dry naturally, and then biomass was measured. The straw was then separated into stems and leaves, dried in a 75 °C oven, ground using a plant grinder, and stored in sealed bags for future use. Twenty rice grains were dehulled into rice husks and brown rice, which were placed in envelope bags and dried in a 75 °C oven until they reached a constant weight for subsequent heavy metal content analysis. Simultaneously, fresh soil samples were transported to the laboratory as soon as possible to extract and measure soil NH_4_^+^-N and NO_3_^−^-N content. The remaining soil samples were naturally air-dried, ground, sieved through 20-mesh and 100-mesh sieves, and sealed for storage, to be used for subsequent analysis of soil physicochemical properties and soil Cd speciation.

### 2.4. Soil and Plant Sample Analysis

Soil analysis indicators include: pH, available phosphorus (AP), NH_4_^+^-N, NO_3_^−^-N, organic matter (SOM), total nitrogen (TN), dissolved organic carbon (DOC), water-soluble Cd, and different chemical forms of Cd (acid-extractable, reducible, oxidizable, residual). Soil chemical properties were analyzed following the methods outlined in “Soil Agricultural Chemical Analysis Methods” edited by Lu Rukun [27]. Soil pH was measured using the water–soil suspension method (water-to-soil ratio of 2.5:1), with a pH meter (F2-Standard, Mettler Toledo, Shanghai, China). Available phosphorus was extracted with 0.5 M sodium bicarbonate solution, and the mixture was shaken at 180 rpm on a reciprocating shaker for 30 min. The phosphorus concentration was determined using the molybdenum-blue colorimetric method and measured with a micro plate spectrophotometer (Infinite 200 PRO, Tecan, Männedorf, Switzerland) with absorbance at 700 nm. Soil NH_4_^+^-N and NO_3_^−^-N were extracted from 5 g fresh soil using 1 M potassium chloride (KCl) solution by shaking for 1 h. The extract was analyzed quantitatively using a continuous flow analyzer (San^++^, Skalar, Breda, The Netherlands). Soil organic matter content was determined using the potassium dichromate oxidation-ferrous sulfate titration method. Total nitrogen in soil was determined by high-temperature combustion in an elemental analyzer (Vario EL CUBE, Elementar, Langenselbold, Germany). Dissolved organic carbon (DOC) was measured by taking 10.0 g of air-dried soil, adding 25 mL of double-distilled water, and shaking the mixture at 180 rpm on a reciprocating shaker for 1 h. The mixture was then centrifuged at 4000 rpm for 10 min, and the supernatant was filtered through a 0.45 µm filter membrane to obtain the water-extracted solution. The DOC content was measured using a TOC analyzer (Multi N/C3100, Analytik Jena, Jena, Germany). Water-soluble Cd and the different chemical forms of Cd (acid-extractable, reducible, oxidizable, residual) in soil were determined using the BCR sequential extraction method [28]. After sequential extraction, Cd content in each fraction was measured. The various plant tissues of rice plants were digested with concentrated nitric acid, appropriately diluted, and then Cd and Mn were quantitatively determined using an inductively coupled plasma-mass spectrometer iCAP RQ (Thermo Fisher Scientific, Waltham, MA, USA), as well as the soil Cd fractions. All measurements, including soil chemical properties, Cd speciation, and Cd and Mn concentrations in plant samples, were expressed on a dry weight basis.

### 2.5. Data Processing

Initial data and calculation were performed using Excel 2016. One-way analysis of variance (ANOVA) was conducted using IBM SPSS Statistics 26.0 to analyze differences in soil physicochemical properties and Cd speciation between different treatments. The Levene’s test was used for testing the homogeneity of variances, and Duncan’s multiple range test was used for post hoc comparisons. Bar charts of means and standard errors were plotted using Origin 2021. The Pearson correlation coefficient and significance were calculated using the “linkET” package in R4.5.0, and the correlation results were visualized as a heatmap using the “corrplot” package (version 0.95).

## 3. Results

### 3.1. Soil Chemical Properties and Cd Speciation Distribution

Compared to the control group (CK), all treatment groups significantly increased soil pH, as shown in Table 2. Among them, the KMnO_4_-enriched biochar treatments (KM1BC2 and KM2BC2) showed the most noticeable increase in pH. Compared to CK, except for BC1 and M1BC1, most of the biochar and manganese-enriched biochar treatment groups also showed an increase in soil organic matter content, which reached a statistically significant level. However, biochar and manganese-enriched biochar treatments did not significantly affect the content of AP, NH_4_^+^-N, NO_3_^−^-N, TN, or DOC (Table 2).

In terms of soil Cd speciation analysis (Table 2), compared to the CK group, both ordinary biochar and manganese-enriched biochar treatments significantly reduced the content of water-soluble Cd, reducible Cd, and oxidizable Cd, indicating that biochar treatments can effectively reduce the mobile and potentially bioavailable Cd fractions in soil. Among them, the KM1BC2 group showed the greatest reduction in water-soluble and reducible Cd, while the M2BC2 group had the lowest content of oxidizable Cd. Except for BC2 and KM1BC1, no significant differences in acid-extractable Cd content were observed across the other treatment groups. However, except for BC1, all other treatment groups had higher residual Cd content than the control group, and manganese-enriched biochar groups generally had higher residual Cd content than the ordinary biochar groups. There were slight differences in total soil Cd content among the treatment groups, but the overall change was minimal, suggesting that biochar and its manganese modifications mainly affect the distribution and mobility of Cd speciation rather than the total Cd content in the soil.

### 3.2. Effects of Different Biochar Treatments on Rice Biomass

**Figure 1:**
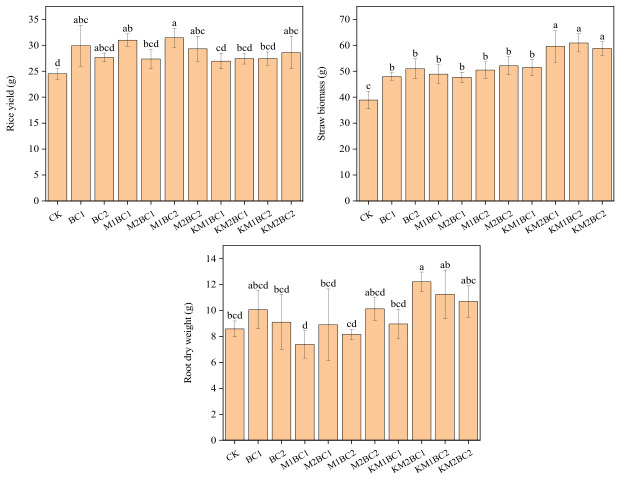
Effects of Ordinary Biochar and Manganese-Enriched Biochar on the Dry Weight of Various Rice Tissues. Different lowercase letters above the bars indicate significant differences among treatments based on Duncan’s test at *p* < 0.05. CK—a control without biochar; BC1—0.5% unenriched biochar; BC2—1.0% unenriched biochar; M1BC1—MnCl_2_-enriched biochar (0.5% addition rate, 27.5 mg Mn/g); M2BC1—MnCl_2_-enriched biochar (0.5% addition rate, 55 mg Mn/g); M1BC2—MnCl_2_-enriched biochar (1% addition rate, 27.5 mg Mn/g); M2BC2—MnCl_2_-enriched biochar (1% addition rate, 55 mg Mn/g); KM1BC1—KMnO_4_-enriched biochar (0.5% addition rate, 27.5 mg Mn/g); KM2BC1—KMnO_4_-enriched biochar (0.5% addition rate, 55 mg Mn/g); KM1BC2—KMnO_4_-enriched biochar (1% addition rate, 27.5 mg Mn/g); KM2BC2—KMnO_4_-enriched biochar (1% addition rate, 55 mg Mn/g).

As shown in Figure 1, compared to the CK treatment, both ordinary biochar and manganese-enriched biochar treatments promoted an increase in rice biomass. The ordinary biochar treatment increased rice yield by approximately 12.77–21.95%, while the manganese-enriched biochar treatment increased rice yield by 9.90–28.29%. Although Mn-enriched biochar effectively reduced Cd bioavailability, its effect on rice yield was comparable to that of conventional biochar. In terms of rice straw biomass, ordinary biochar, MnCl_2_-enriched biochar, and KMnO_4_-enriched biochar all significantly increased biomass compared to the CK group, with increases of 23.22–31.01%, 22.56–33.92%, and 32.37–56.52%, respectively. Compared to the changes in rice grain yield or straw biomass, the differences in root dry weight among the different treatment groups were relatively small and did not show a consistent significant pattern.

### 3.3. Accumulation of Mn and Cd in Different Rice Tissues and Characteristics of Root Uptake Efficiency

**Figure 2:**
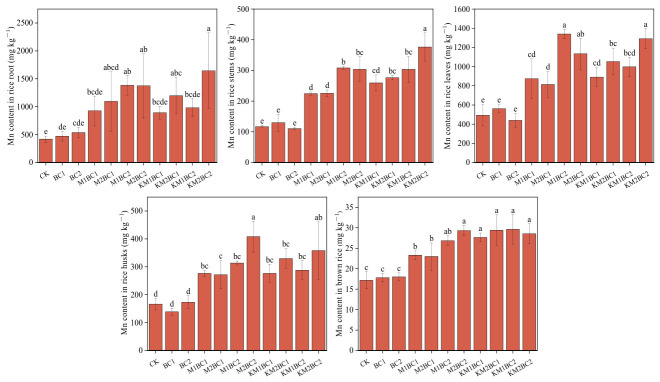
Changes in Mn Content in Various Rice Tissues Under Different Treatments. Different lowercase letters above the bars indicate significant differences among treatments based on Duncan’s test at *p* < 0.05. CK—a control without biochar; BC1—0.5% unenriched biochar; BC2—1.0% unenriched biochar; M1BC1—MnCl_2_-enriched biochar (0.5% addition rate, 27.5 mg Mn/g); M2BC1—MnCl_2_-enriched biochar (0.5% addition rate, 55 mg Mn/g); M1BC2—MnCl_2_-enriched biochar (1% addition rate, 27.5 mg Mn/g); M2BC2—MnCl_2_-enriched biochar (1% addition rate, 55 mg Mn/g); KM1BC1—KMnO_4_-enriched biochar (0.5% addition rate, 27.5 mg Mn/g); KM2BC1—KMnO_4_-enriched biochar (0.5% addition rate, 55 mg Mn/g); KM1BC2—KMnO_4_-enriched biochar (1% addition rate, 27.5 mg Mn/g); KM2BC2—KMnO_4_-enriched biochar (1% addition rate, 55 mg Mn/g).

As shown in Figure 2, after the application of manganese-enriched biochar, the Mn content in various rice organs—including roots, stems, leaves, rice husks, and brown rice—was significantly higher than that in the CK and unenriched biochar treatments. All manganese-enriched biochar treatment groups showed a pronounced trend of increased Mn accumulation, indicating that manganese-enriched biochar can significantly enhance rice uptake of Mn and its translocation to above-ground tissues.

**Figure 3:**
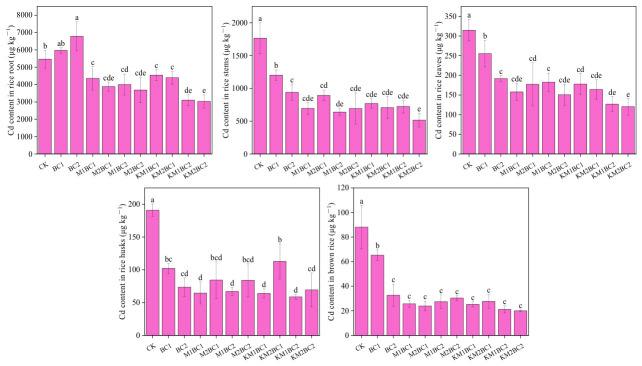
Changes in Cd Content in Various Rice Tissues Under Different Treatments. Different lowercase letters above the bars indicate significant differences among treatments based on Duncan’s test at *p* < 0.05. CK—a control without biochar; BC1—0.5% unenriched biochar; BC2—1.0% unenriched biochar; M1BC1—MnCl_2_-enriched biochar (0.5% addition rate, 27.5 mg Mn/g); M2BC1—MnCl_2_-enriched biochar (0.5% addition rate, 55 mg Mn/g); M1BC2—MnCl_2_-enriched biochar (1% addition rate, 27.5 mg Mn/g); M2BC2—MnCl_2_-enriched biochar (1% addition rate, 55 mg Mn/g); KM1BC1—KMnO_4_-enriched biochar (0.5% addition rate, 27.5 mg Mn/g); KM2BC1—KMnO_4_-enriched biochar (0.5% addition rate, 55 mg Mn/g); KM1BC2—KMnO_4_-enriched biochar (1% addition rate, 27.5 mg Mn/g); KM2BC2—KMnO_4_-enriched biochar (1% addition rate, 55 mg Mn/g).

As shown in Figure 3, in the unenriched biochar treatment group, the Cd content in rice roots was slightly higher than that in the CK group, but the manganese-enriched biochar treatment significantly reduced the Cd content in rice roots. The Cd content in rice straw, leaves, husks, and brown rice showed a consistent pattern. Compared to the CK group, the unenriched biochar treatment (BC1) significantly reduced Cd content in all tissues, and at the higher application rate (BC2), Cd content was further reduced. In contrast, Cd content in rice straw, leaves, husks, and brown rice in the manganese-enriched biochar treatments with either low or high manganese content showed no significant differences compared to the BC2 group.

**Figure 4:**
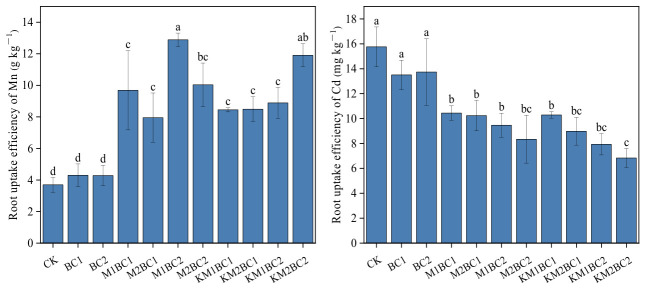
Changes in Mn and Cd Uptake Efficiency of Rice Under Different Treatments. Different lowercase letters above the bars indicate significant differences among treatments based on Duncan’s test at *p* < 0.05. CK—a control without biochar; BC1—0.5% unenriched biochar; BC2—1.0% unenriched biochar; M1BC1—MnCl_2_-enriched biochar (0.5% addition rate, 27.5 mg Mn/g); M2BC1—MnCl_2_-enriched biochar (0.5% addition rate, 55 mg Mn/g); M1BC2—MnCl_2_-enriched biochar (1% addition rate, 27.5 mg Mn/g); M2BC2—MnCl_2_-enriched biochar (1% addition rate, 55 mg Mn/g); KM1BC1—KMnO_4_-enriched biochar (0.5% addition rate, 27.5 mg Mn/g); KM2BC1—KMnO_4_-enriched biochar (0.5% addition rate, 55 mg Mn/g); KM1BC2—KMnO_4_-enriched biochar (1% addition rate, 27.5 mg Mn/g); KM2BC2—KMnO_4_-enriched biochar (1% addition rate, 55 mg Mn/g).

We further assessed the absorption efficiency of manganese or cadmium by dividing the total metal content (Mn or Cd) measured in the entire plant by the corresponding root dry weight to calculate the root uptake efficiency of Mn or Cd (Figure 4). The results showed that the root absorption efficiency of Mn or Cd in the unenriched biochar treatment group was not significantly different from that in the CK group. However, after applying manganese-enriched biochar, the rice roots showed a significant increase in Mn absorption efficiency, while Cd absorption efficiency was significantly reduced.

### 3.4. Mn and Cd Content, Uptake Efficiency in Different Rice Tissues, and Their Correlation with Soil Properties and Cd Speciation

**Figure 5:**
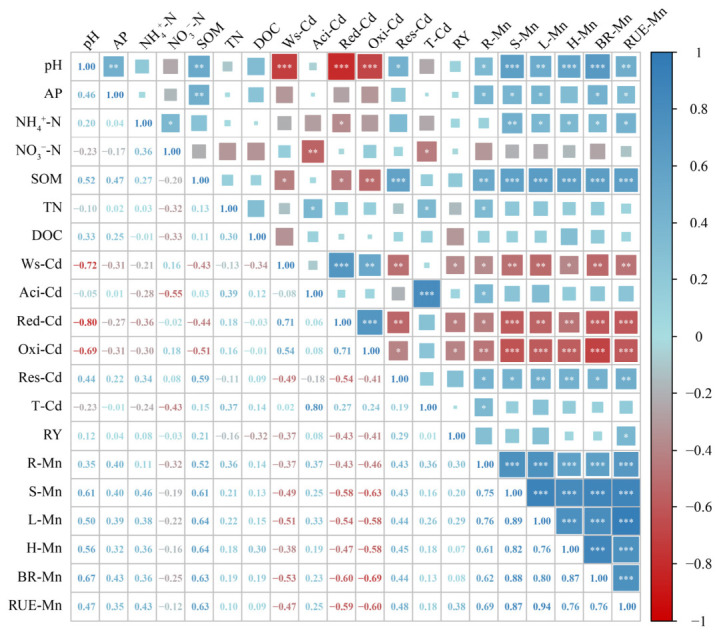
Correlation Analysis of Soil Chemical Properties, Cadmium Speciation, Rice Yield, and Mn Uptake Efficiency. Pearson correlation coefficient: *, *p* < 0.05; **, *p* < 0.01; ***, *p* < 0.001. The size and color intensity of the squares represent the magnitude of the correlation coefficient, with larger and darker squares indicating stronger relationships. AP—available phosphorus; SOM—soil organic matter; TN—total nitrogen; DOC—dissolved organic carbon; Ws-Cd—water-soluble cadmium; Aci-Cd—acid-extractable cadmium; Red-Cd—reducible cadmium; Oxi-Cd—oxidizable cadmium; Res-Cd—residual cadmium; T-Cd—total cadmium; RY—rice yield; R-Mn—manganese content in rice root; S-Mn—manganese content in rice stems; L-Mn—manganese content in rice leaves; H-Mn—manganese content in rice husks; BR-Mn—manganese content in brown rice; RUE-Mn—Root uptake efficiency of manganese.

The correlation analysis results show that soil pH and organic matter were significantly negatively correlated with water-soluble Cd, reducible Cd, and oxidizable Cd, while they were positively correlated with residual Cd (Figure 5). The accumulation and uptake efficiency of manganese in different rice tissues (roots, stems, leaves, husks, and brown rice) were significantly positively correlated with soil pH and organic matter, while they were significantly negatively correlated with water-soluble Cd, reducible Cd, and oxidizable Cd in soil, and significantly positively correlated with residual Cd (Figure 5).

**Figure 6:**
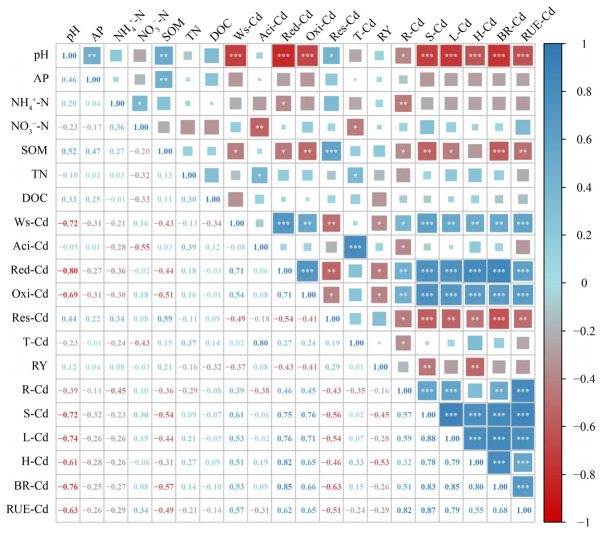
Correlation Analysis of Soil Chemical Properties, Cadmium Speciation, Rice Yield, and Cd Uptake Efficiency. Pearson correlation coefficient: *, *p* < 0.05; **, *p* < 0.01; ***, *p* < 0.001. The size and color intensity of the squares represent the magnitude of the correlation coefficient, with larger and darker squares indicating stronger relationships. AP—available phosphorus; SOM—soil organic matter; TN—total nitrogen; DOC—dissolved organic carbon; Ws-Cd—water-soluble cadmium; Aci-Cd—acid-extractable cadmium; Red-Cd—reducible cadmium; Oxi-Cd—oxidizable cadmium; Res-Cd—residual cadmium; T-Cd—total cadmium; RY—rice yield; R-Cd—cadmium content in rice root; S-Cd—cadmium content in rice stems; L-Cd—cadmium content in rice leaves; H-Cd—cadmium content in rice husks; BR-Cd—cadmium content in brown rice; RUE-Cd—Root uptake efficiency of cadmium.

As shown in Figure 6, soil pH and organic matter were significantly negatively correlated with the accumulation and uptake efficiency of Cd in rice tissues (roots, stems, leaves, husks, and brown rice). However, water-soluble Cd, reducible Cd, and oxidizable Cd were significantly positively correlated with Cd content and Cd uptake efficiency in rice roots, stems, leaves, husks, and brown rice, while residual Cd was negatively correlated with Cd content and Cd uptake efficiency in all rice tissues (Figure 6).

**Figure 7:**
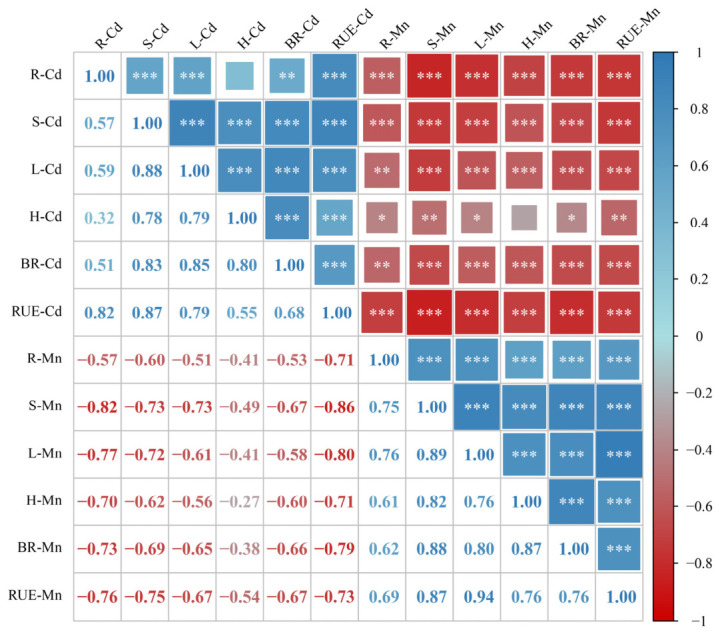
Correlation Analysis of Mn and Cd Accumulation and Uptake Efficiency in Different Rice Tissues. Pearson correlation coefficient: *, *p* < 0.05; **, *p* < 0.01; ***, *p* < 0.001. The size and color intensity of the squares represent the magnitude of the correlation coefficient, with larger and darker squares indicating stronger relationships. R-Cd—cadmium content in rice root; S-Cd—cadmium content in rice stems; L-Cd—cadmium content in rice leaves; H-Cd—cadmium content in rice husks; BR-Cd—cadmium content in brown rice; RUE-Cd—Root uptake efficiency of cadmium; R-Mn—manganese content in rice root; S-Mn—manganese content in rice stems; L-Mn—manganese content in rice leaves; H-Mn—manganese content in rice husks; BR-Mn—manganese content in brown rice; RUE-Mn—Root uptake efficiency of manganese.

Correlation matrix analysis showed that the Cd content in rice roots, stems, leaves, and husks was significantly positively correlated with the Cd content in brown rice and Cd uptake efficiency. At the same time, the Cd content and Cd uptake efficiency in rice roots, stems, leaves, husks, and brown rice were significantly negatively correlated with the Mn content and Mn uptake efficiency in all rice tissues (Figure 7).

## 4. Discussion

The results of this study show that both unenriched biochar and Mn-enriched biochar significantly increased soil pH and organic matter content, and promoted the transformation of Cd speciation from soluble, reducible, and oxidizable forms toward a stable residual form. These changes indicate that the reduction in Cd bioavailability was primarily governed by modifications in the soil chemical environment. A rise in soil pH is widely recognized as a key factor reducing Cd mobility because it decreases Cd solubility, enhances negative charges on soil surfaces, and favors Cd adsorption or precipitation [29]. In our study, the strong negative correlations between soil pH and water-soluble, reducible, and oxidizable Cd, together with the positive correlation between pH and residual Cd, further support this mechanism. The observed increase in SOM is likely attributed to the direct input of biochar rather than the formation of stabilized humus, especially given the relatively short duration of the experiment. Consistently, no significant increase in DOC was observed, further indicating that humification processes were limited during the experimental period. Biochar itself is characterized by a high specific surface area, abundant oxygen-containing functional groups (e.g., carboxyl and hydroxyl), a well-developed pore structure, and high cation exchange capacity (CEC), which enable it to immobilize Cd^2+^ through mechanisms such as surface complexation, ion exchange, or precipitation, thereby markedly reducing Cd mobility and its bioavailability in soil [30,31]. Therefore, the reduction in Cd uptake under biochar treatments can be explained by the combined effects of pH increase, shifts in Cd fractionation, and the sorption capacity of biochar-derived carbon. This interpretation is also consistent with the reduced Cd accumulation observed in roots, shoots, husks, and brown rice, as well as the lower root Cd uptake efficiency under biochar-amended treatments.

Cadmium (Cd), though not an essential element for plant growth and development, has been shown to enter rice through the metal transporter OsNRAMP5, which primarily serves to transport manganese (Mn). Knockout or loss-of-function mutants of OsNRAMP5 exhibit drastically reduced uptake and accumulation of both Mn and Cd in roots and shoots, underscoring that OsNRAMP5 is a major pathway for Cd entry into the rice plant body [17,32]. Except for rice, accumulating evidence indicates that NRAMP5 homologues in other plant species also mediate Cd uptake and transport [33,34]. Recent studies have exploited the competitive/antagonistic interaction between Mn and Cd to mitigate Cd uptake and accumulation in plants. For example, studies have shown that exogenous Mn application to rice seedlings can significantly reduce Cd uptake and translocation, likely because Mn competes with Cd at root uptake sites and suppresses Cd transporter expression such as OsNRAMP5 and OsIRT1 [35,36]. In agreement with this mechanism, our correlation analysis showed that Mn concentrations in different rice tissues and root Mn uptake efficiency were significantly negatively correlated with Cd concentrations in the corresponding tissues and with root Cd uptake efficiency. This pattern suggests that higher Mn availability in the rhizosphere and plant system may have restricted Cd entry and translocation through antagonistic interactions at the root uptake level. In addition to competing with Cd for plant uptake, Mn^2+^ inputs can enhance the formation of Mn oxides, which can immobilize heavy metals in soil through adsorption, surface complexation, and the formation of stable hydroxide precipitates or nodular structures, ultimately reducing Cd bioavailability [22,37,38,39]. Thus, the reduction in Cd under Mn-enriched biochar treatments is likely attributed to the combined effects of biochar surface adsorption and Mn-mediated mechanisms, including enhanced Cd immobilization in soil and competitive inhibition of Cd uptake by plants. However, this effect should not be overstated. In some cases, the differences between Mn-enriched biochar and unenriched biochar were not statistically significant, particularly at the higher biochar application rate. Therefore, the advantage of Mn enrichment is better reflected as an enhancement of remediation efficiency under specific conditions, rather than a universally superior effect across all treatments. A related point concerns the positive correlation observed between SOM and plant Mn content. Based on our results, conventional biochar did not markedly increase Mn concentrations in plant tissues relative to the control, whereas Mn-enriched biochar clearly did. Therefore, we propose that the positive SOM–Mn relationship is primarily a treatment-driven effect associated with Mn-enriched biochar, which simultaneously increased SOM through biochar input and Mn availability through Mn loading, rather than indicating a direct causal role of SOM itself in promoting Mn uptake.

Interestingly, we found that low application rate of Mn-enriched biochar (M1BC1, M2BC1, KM1BC1, KM2BC1) resulted in a reduction in Cd accumulation in different rice tissues comparable to those achieved with a high-dose unenriched biochar (BC2) despite the biochar application rate being halved. This result is particularly meaningful from an application perspective. Biochar is typically produced by pyrolyzing biomass (e.g., wood or crop residues) under oxygen-limited conditions at around 300–700 °C, resulting in a stable, porous carbon-rich solid [40]. The production of biochar requires substantial energy input for heating and maintaining pyrolysis conditions, and the costs are estimated at between US $571 and $1455 per ton of biochar, underscoring the considerable resource demand and economic burden associated with biochar production [41]. Given a surface-soil bulk density of 1.28 g cm^−3^ [42,43], the mass of soil per hectare of topsoil can be calculated as approximately 2.56 × 10^3^ t ha^−1^. Therefore, a high-dose unenriched biochar (1%) to remediate one hectare of Cd-polluted soil would require about 25.6 t biochar (≈US $14,618–37,248). However, a low application rate of Mn-enriched biochar (0.5%) would require only 12.8 t of biochar (≈US $7309–18,624), with an additional manganese chloride cost of approximately US $1450 (based on an application rate of 1.27 t ha^−1^ industrial-grade MnCl_2_·4H_2_O priced at RMB 8000 t^−1^), while achieving a remediation effectiveness equivalent to that of high-dose unenriched biochar and resulting in a substantially lower overall remediation cost. Both conventional biochar and Mn-enriched biochar generally improved rice biomass and yield relative to the control, but the differences between the two types of biochar were often not statistically significant. This indicates that Mn enrichment did not further enhance yield, and its primary benefit lies in reducing Cd accumulation, especially in edible tissues, rather than in promoting biomass production. Therefore, the practical value of Mn-enriched biochar is best understood in terms of achieving similar remediation performance with a reduced biochar input, which may lower material demand and overall remediation cost. It should also be noted that this study was based on a short-term pot experiment under controlled conditions, which may not fully represent the complexity of field environments. Factors such as soil heterogeneity, environmental variability, microbial dynamics, and long-term stabilization processes were not considered. Therefore, while the present results provide strong evidence for the potential of Mn-enriched biochar as a more efficient remediation strategy, future studies should focus on long-term field trials to evaluate the stability of Cd immobilization, environmental adaptability, and potential effects on micronutrient availability under realistic agricultural conditions.

## 5. Conclusions

This study demonstrated that both unenriched biochar and Mn-enriched biochar effectively reduced Cd bioavailability and accumulation in rice grown in Cd-contaminated paddy soil. The reduction in Cd uptake was closely associated with increased soil pH and organic matter content, as well as the transformation of Cd from labile fractions (water-soluble, reducible, and oxidizable Cd) to more stable residual forms. Correlation analysis further confirmed that soil pH and organic matter were significantly negatively correlated with bioavailable Cd fractions and Cd accumulation in rice tissues, while positively correlated with residual Cd. Mn-enriched biochar exhibited an additional effect by significantly enhancing Mn accumulation and uptake efficiency in rice, while simultaneously reducing Cd uptake efficiency. The significant negative correlations between Mn content (and Mn uptake efficiency) and Cd accumulation across different rice tissues indicate a strong antagonistic interaction between Mn and Cd at the plant level. Notably, Mn-enriched biochar achieved Cd reduction effects in rice tissues comparable to those of higher-dose conventional biochar, particularly at lower application rates, suggesting improved remediation efficiency. Overall, the results indicate that Mn-enriched biochar reduces Cd accumulation through combined effects of soil chemical modification and Mn-related interactions in the soil–plant system, and represents a potentially cost-effective strategy for mitigating Cd contamination in paddy soils.

## Figures and Tables

**Table 1:** Experimental design of biochar treatments with different Mn modification methods and application rates.

Treatment Groups	Biochar Type	Addition Rate (%)	Mn Content (mg Mn/g BC)
CK	a control without biochar	0	0
BC1	unenriched biochar	0.5	0
BC2	unenriched biochar	1.0	0
M1BC1	MnCl_2_-enriched biochar	0.5	27.5
M2BC1	MnCl_2_-enriched biochar	0.5	55.0
M1BC2	MnCl_2_-enriched biochar	1.0	27.5
M2BC2	MnCl_2_-enriched biochar	1.0	55.0
KM1BC1	KMnO_4_-enriched biochar	0.5	27.5
KM2BC1	KMnO_4_-enriched biochar	0.5	55.0
KM1BC2	KMnO_4_-enriched biochar	1.0	27.5
KM2BC2	KMnO_4_-enriched biochar	1.0	55.0

**Table 2:** Basic Physicochemical Properties of Soil and Cadmium Speciation.

Soil Chemical Properties and Cd Speciation Distribution	CK	BC1	BC2	M1BC1	M2BC1	M1BC2	M2BC2	KM1BC1	KM2BC1	KM1BC2	KM2BC2
pH	6.59 ± 0.09 ^e^	6.80 ± 0.02 ^d^	6.94 ± 0.04 ^abc^	6.81 ± 0.06 ^d^	6.83 ± 0.02 ^d^	6.88 ± 0.01 ^cd^	6.91 ± 0.02 ^bcd^	6.93 ± 0.01 ^abc^	6.99 ± 0.09 ^ab^	7.02 ± 0.01 ^a^	7.04 ± 0.12 ^a^
AP (mg kg^−1^)	15.26 ± 0.33 ^cde^	15.42 ± 0.19 ^bcde^	17.03 ± 1.98 ^abcd^	14.48 ± 0.41 ^de^	14.42 ± 0.99 ^e^	18.16 ± 2.71 ^a^	16.62 ± 0.83 ^abcde^	17.83 ± 1.12 ^abc^	16.77 ± 0.95 ^abcde^	17.15 ± 1.44 ^abc^	17.88 ± 1.62 ^ab^
NH_4_^+^-N (mg kg^−1^)	7.84 ± 0.21 ^ab^	8.74 ± 1.33 ^ab^	7.42 ± 0.15 ^b^	8.56 ± 0.86 ^ab^	8.60 ± 1.22 ^ab^	8.83 ± 1.07 ^ab^	9.14 ± 0.35 ^a^	9.35 ± 0.48 ^a^	8.43 ± 0.64 ^ab^	8.91 ± 0.65 ^ab^	8.79 ± 1.06 ^ab^
NO_3_^−^-N (mg kg^−1^)	20.52 ± 1.06 ^a^	20.89 ± 0.97 ^a^	19.96 ± 1.07 ^a^	21.04 ± 0.97 ^a^	20.61 ± 2.00 ^a^	20.40 ± 0.68 ^a^	20.86 ± 0.96 ^a^	20.99 ± 0.78 ^a^	19.30 ± 0.76 ^a^	20.12 ± 0.19 ^a^	19.25 ± 1.02 ^a^
SOM (g kg^−1^)	29.75 ± 1.24 ^fg^	28.95 ± 2.21 ^g^	33.02 ± 0.49 ^cde^	31.12 ± 0.93 ^ef^	32.37 ± 0.77 ^de^	38.33 ± 0.61 ^a^	34.78 ± 0.88 ^bc^	33.50 ± 1.47 ^cd^	35.99 ± 1.55 ^b^	32.62 ± 0.80 ^cde^	33.76 ± 0.91 ^cd^
TN (g kg^−1^)	0.61 ± 0.05 ^a^	0.57 ± 0.03 ^a^	0.56 ± 0.03 ^a^	0.55 ± 0.02 ^a^	0.60 ± 0.07 ^a^	0.59 ± 0.01 ^a^	0.59 ± 0.01 ^a^	0.57 ± 0.02 ^a^	0.56 ± 0.01 ^a^	0.59 ± 0.01 ^a^	0.61 ± 0.01 ^a^
DOC (mg kg^−1^)	141.00 ± 4.63 ^a^	143.67 ± 8.33 ^a^	146.67 ± 8.57 ^a^	139.67 ± 11.03 ^a^	146.75 ± 10.19 ^a^	141.33 ± 11.21 ^a^	142.58 ± 7.26 ^a^	141.83 ± 4.63 ^a^	142.75 ± 9.76 ^a^	144.00 ± 9.69 ^a^	154.25 ± 18.56 ^a^
Ws-Cd (μg kg^−1^)	2.28 ± 0.92 ^a^	0.91 ± 0.17 ^bc^	1.27 ± 0.30 ^bc^	1.13 ± 0.11 ^bc^	1.16 ± 0.40 ^bc^	0.74 ± 0.42 ^bc^	1.24 ± 0.21 ^bc^	1.37 ± 0.15 ^b^	0.75 ± 0.40 ^bc^	0.59 ± 0.13 ^c^	0.68 ± 0.38 ^bc^
Aci-Cd (μg kg^−1^)	807.15 ± 12.51 ^a^	776.40 ± 22.52 ^abc^	768.52 ± 25.66 ^bc^	788.01 ± 11.06 ^abc^	780.40 ± 21.38 ^abc^	802.06 ± 18.32 ^ab^	786.17 ± 25.88 ^abc^	757.68 ± 16.98 ^c^	797.12 ± 8.33 ^ab^	808.49 ± 26.00 ^a^	808.61 ± 4.89 ^a^
Red-Cd (μg kg^−1^)	218.93 ± 7.99 ^a^	186.53 ± 3.08 ^b^	185.36 ± 8.67 ^b^	178.62 ± 3.75 ^bc^	183.60 ± 7.55 ^b^	178.20 ± 6.83 ^bc^	179.29 ± 7.60 ^bc^	180.61 ± 3.04 ^bc^	179.59 ± 5.60 ^bc^	168.92 ± 8.18 ^c^	175.24 ± 5.82 ^bc^
Oxi-Cd (μg kg^−1^)	25.44 ± 0.65 ^a^	22.98 ± 0.90 ^b^	22.90 ± 1.32 ^bc^	22.03 ± 0.79 ^bcd^	22.84 ± 1.26 ^bc^	21.88 ± 0.59 ^bcd^	20.89 ± 0.46 ^d^	21.75 ± 0.73 ^bcd^	21.45 ± 0.43 ^bcd^	21.27 ± 0.36 ^cd^	22.21 ± 1.24 ^bcd^
Res-Cd (μg kg^−1^)	104.21 ± 8.20 ^d^	120.14 ± 7.40 ^cd^	132.23 ± 7.47 ^bc^	135.95 ± 9.35 ^abc^	155.41 ± 24.84 ^a^	145.78 ± 9.22 ^ab^	141.99 ± 7.68 ^abc^	140.35 ± 13.16 ^abc^	153.21 ± 10.10 ^ab^	131.26 ± 11.73 ^bc^	134.29 ± 8.92 ^abc^
T-Cd (μg kg^−1^)	1158.01 ± 12.64 ^a^	1106.97 ± 15.60 ^bc^	1110.28 ± 31.79 ^bc^	1125.74 ± 24.16 ^abc^	1143.42 ± 15.57 ^abc^	1148.66 ± 33.70 ^ab^	1129.58 ± 22.57 ^abc^	1101.75 ± 25.18 ^c^	1152.13 ± 21.62 ^a^	1130.53 ± 8.00 ^abc^	1141.02 ± 8.48 ^abc^

Data are presented as means ± SD (n = 3). Values in the same row followed by different lowercase letters are significantly different (*p* < 0.05, Duncan’s test). CK—a control without biochar; BC1—0.5% unenriched biochar; BC2—1.0% unenriched biochar; M1BC1—MnCl_2_-enriched biochar (0.5% addition rate, 27.5 mg Mn/g); M2BC1—MnCl_2_-enriched biochar (0.5% addition rate, 55 mg Mn/g); M1BC2—MnCl_2_-enriched biochar (1% addition rate, 27.5 mg Mn/g); M2BC2—MnCl_2_-enriched biochar (1% addition rate, 55 mg Mn/g); KM1BC1—KMnO_4_-enriched biochar (0.5% addition rate, 27.5 mg Mn/g); KM2BC1—KMnO_4_-enriched biochar (0.5% addition rate, 55 mg Mn/g); KM1BC2—KMnO_4_-enriched biochar (1% addition rate, 27.5 mg Mn/g); KM2BC2—KMnO_4_-enriched biochar (1% addition rate, 55 mg Mn/g); AP—available phosphorus; SOM—soil organic matter; TN—total nitrogen; DOC—dissolved organic carbon; Ws-Cd—water-soluble cadmium; Aci-Cd—acid-extractable cadmium; Red-Cd—reducible cadmium; Oxi-Cd—oxidizable cadmium; Res-Cd—residual cadmium; T-Cd—total Cadmium.

## Data Availability

The original contributions presented in this study are included in the article. Further inquiries can be directed to the corresponding author.

## Abbreviations

The following abbreviations are used in this manuscript:

Cd	Cadmium
Mn	Manganese
C	Carbon
H	Hydrogen
O	Oxygen
N	Nitrogen
P	Phosphorus
K	Potassium
Ca	Calcium
Na	Sodium
Mg	Magnesium
As	Arsenic
TCLP	Toxicity Characteristic Leaching Procedure
AP	Available phosphorus
SOM	Soil organic matter
TN	Total nitrogen
DOC	Dissolved organic carbon
ICP-MS	Inductively coupled plasma mass spectrometry
TOC	Total organic carbon
BCR	Community Bureau of Reference
ANOVA	Analysis of variance
Ws-Cd	Water-soluble cadmium
Aci-Cd	Acid-extractable cadmium
Red-Cd	Reducible cadmium
Oxi-Cd	Oxidizable cadmium
Res-Cd	Residual cadmium
T-Cd	Total cadmium
SD	Standard deviation
RY	Rice yield
R-Mn	Manganese content in rice root
S-Mn	Manganese content in rice stems
L-Mn	Manganese content in rice leaves
H-Mn	Manganese content in rice husks
BR-Mn	Manganese content in brown rice
RUE-Mn	Root uptake efficiency of manganese
R-Cd	Cadmium content in rice root
S-Cd	Cadmium content in rice stems
L-Cd	Cadmium content in rice leaves
H-Cd	Cadmium content in rice husks
BR-Cd	Cadmium content in brown rice
RUE-Cd	Root uptake efficiency of cadmium
CEC	Cation exchange capacity
